# Subhealth Risk Perception Scale: Development and Validation of a New Measure

**DOI:** 10.1155/2022/9950890

**Published:** 2022-01-10

**Authors:** Jiangjie Sun, Xueli Jiang, Yufei Gao, Chengsen He, Mingxin Wang, Xiaohua Wang, Zhengzhi Yu, Xiaomao Si, Xiuyan Shi, Liping Zhang

**Affiliations:** ^1^Health Management College, Anhui Medical University, Hefei 230032, China; ^2^Clinical Medical College, Anhui Medical University, Hefei 230031, China; ^3^School of Marxism Studies, Anhui Medical University, Hefei, Anhui 230032, China; ^4^Medical Examination Center, The First Affiliated Hospital of Anhui Medical University, Hefei 230022, China; ^5^Nursing Department, The First Affiliated Hospital of Wannan Medical College, Wuhu 241001, China; ^6^Department of General Surgery, The Traditional Chinese Medicine Hospital of Nanling County Wuhu City, Wuhu 241300, China; ^7^Nanjing Prevention and Treatment Center for Occupational Diseases, Nanjing 231000, China

## Abstract

**Background:**

To develop an individual's physical subhealth risk perception scale and evaluate its reliability and validity, so as to provide a measurement tool for individual physical health risk.

**Methods:**

A questionnaire on the perception risk of physical subhealth was developed. Using a random sampling method, 785 people in the Anhui provincial physical examination centre were selected as the research participants. Of the questionnaires returned, 770 were valid, giving an effective rate of 98%. Firstly, the Pearson correlation coefficient method was used to study the correlation of 35 items in the initial scale, and then, polychoric factor structure analysis was carried out by using the Pratt *D* matrix to optimize the item structure. The Cronbach'*α* coefficient method was used to test the internal consistency reliability, and a structural equation model was used to explore the construct validity of the scale. The discriminant validity of the scale was obtained by factor analysis. A general linear model was used to analyse the relationship between the clinical manifestations of physical subhealth and the level of risk perception, and the convergent validity of the scale was evaluated.

**Results:**

All the data of 35 items were significantly correlated at the 0.01 level. The correlation coefficients between a1 and a2, a3 and a4, b1 and b2, b2 and b3, c4 and c5, c5 and c6, c6 and c7, c8 and c9, d1 and d2, d2 and d3, e5 and e6, g1 and g2, g2 and g3, and g2 and g4 were greater than 0.6. The items with correlation coefficients greater than 0.6 were reduced by a Pratt *D* matrix. The resulting physical subhealth risk perception scale covers five factors with a total of 18 items. The Cronbach'*α* coefficient of the scale was 0.889, and the Cronbach'*α* coefficients of the five factors F1-F5 were 0.780, 0.825, 0.801, 0.736, and 0.704, respectively. Structural equation model analysis showed that *χ*^2^/df = 3.43, *p* < 0.001, RMSEA = 0.08, GFI = 0.88, NFI = 0.84, AGFI = 0.84, and CFI = 0.88. Factor analysis showed that factors F1–F5 had significant correlations (*p* < 0.01), and the correlation coefficients were less than the corresponding square root value of AVE. Based on the subhealth clinical manifestations of the participants, the general linear model was used to explore the convergent validity of the scale, and the results indicated that the scale passed the convergent validity test.

**Conclusions:**

We propose a physical subhealth risk perception scale amounting to 18 items, which includes five dimensions: health knowledge (2 items), risk perception (5 items), trust selection (4 items), information channel (4 items), and social groups (3 items). The reliability and validity of the physical subhealth risk perception scale are acceptable. Applying the scale into practice has potential to improve the overall public health level.

## 1. Background

The 5000-year-old fine historical tradition of the Chinese nation has nurtured generations of Chinese people with a simple style of living. In the early years, the living standard of the domestic people was low, coupled with hardworking and the feelings of children's supremacy, the limited economic income was mostly invested in the cultivation of children, lowering the quality of life of the public, and the public subhealth gained little attention, especially in rural areas, and this phenomenon was particularly prominent. In recent years, although the living conditions of the public have improved, the contradiction between the growth of children's education and the lack of family income still exists. Public subhealth problems are serious, young and middle-aged people have been suddenly attacked by serious illnesses, and the disintegration of harmonious families also happens from time to time. Diabetes, hypertension, coronary heart disease, and other chronic diseases account for a large proportion. Therefore, subhealth problems might be worse in China because of the cultural factors.

Subhealth is one of the most difficult problems in modern medicine that endanger human health. As early as the mid-1980s, Professor Berkman, a former soviet scholar, put forward a concept of the subhealth state. He believed that the human body has an intermediate state of nonhealth and nondisease besides the state of health and disease. He called this state “third state” or “gray state” [1]. It was officially defined as “sub-health state” at the 8th Subhealth Symposium in 2001 [2]. Since then, the term “sub-health” has been widely used in the world and has attracted great attention from scholars.

There are many kinds of subhealth symptoms, such as physical fatigue, psychological anxiety and depression, and the decline of social adaptability. The process of body life is the mutual transformation of health, subhealth, and disease. If intervention is not in time, subhealth will lead to the occurrence of diseases [3]. The existence of subhealth not only affects the quality of life and happiness index of individuals but also leads to the decline of social creativity. An increasing number of people are in the subhealth state because of the increasingly fierce competition environment and the double pressure of life and work. The World Health Organization stated that only 5% of the global population were in a complete health state, 20% were in a disease state, and the remaining 75% were in subhealth status in the whole world [4]. As a result, subhealth has become a global public health problem that needs urgent attention and expected to be solved.

In the current research, there is a lack of self-measurement tools to prevent physical subhealth. Therefore, this study developed a physical subhealth risk perception questionnaire and further developed a physical subhealth risk perception scale, which will improve the public's subhealth awareness, enhance the public's enthusiasm for subhealth screening, improve the quality of life of the population, and contribute to the prevention of physical subhealth in the public.

## 2. Literature Review

### 2.1. Subhealth

With the transformation of the biopsychosocial medical model and the implementation of the “Healthy China” strategy, the prevention and treatment of subhealth status has become a medical problem to be solved urgently [5, 6]. The clinical diagnostic criteria and examination methods of modern medicine are ineffective in subhealth problems. Traditional Chinese medicine practitioners use “looking, listening, asking, and feeling” to make a diagnosis, which lacks accuracy in the prevention and treatment of subhealth. Therefore, using a questionnaire or scale to evaluate subhealth status is one of the most commonly used methods [7]. At present, the Self-reporting Inventory developed by Deragotis et al. is one of the more common self-assessment scales [8]. The scale includes 90 items such as emotion, thinking, consciousness, living habits, diet, and sleep. It is mainly used to measure the mental health status of individuals. Other scales for evaluating mental health status include the Self-Rating Anxiety Scale [9], Self-Rating Depression Scale [10], and Eysenck Personality Questionnaire [11]. The above scales focus only on the measurement of mental health and lack the measurement of the individual's physical health. In 2019, Zhao et al. proposed the Cornell Medical Index, whose measurement content includes four parts: physical conditions, family history and anamnesis, general health and habits, and mental symptoms [12]. It can objectively and comprehensively reflect the physical as well as mental problems of the participants, while lacks advance prediction and intervention for physical subhealth. Traditional Chinese Medicine Health Self-evaluation Scale [13] assesses individual health in four dimensions: physical feeling, psychological state, natural adaptability, and social adaptability, combined with the characteristics of traditional Chinese medicine, adding elements such as energy, spirit, sleep, diet, stool, and body fluid. The Short Form Health Questionnaire (SF-36) [14] assesses individual health from three dimensions: exercise status, reasons for exercise restriction, and psychology and introduces the measurement index of physical health into the mental health measurement. Due to the lack of precision of the traditional Chinese medicine measures and the fact that exercise status is only one facet of physical health, both questionnaires have certain shortcomings. The abovementioned scales are diagnostic tools to measure the health of patients and cannot provide early warning in potential subhealth stage.

### 2.2. Risk Perception

Risk perception is used to describe people's attitude towards risk. It is an intuitive judgment and belongs to the category of psychology [15]. In 1960, Professor Bauer of Harvard University first proposed “risk perception” and applied it to the study of consumer behaviour [16]. He believed that all behaviours of consumers have the possibility of producing unpleasant results, and the possibility has its own direct judgment or cognition. Risk perception is one of the core elements of health behavior theories [17], which demonstrated that when people are exposed to risk, risk perception motivates people to stop unhealthy behaviours and adopts healthy behaviours to avoid negative consequences [18]. The level of risk perception affects the behavioral lifestyle of the public [19], while lifestyle is one of the most important factors influencing physical health. We therefore discussed the role of risk perception in physical subhealth from a psychological perspective. Risk perception has a wide range of applications in the medical field. For example, Zhao et al. pointed out that understanding the risk perceptions of cancer patients is a way to facilitate effective clinical decision-making and suggested that appropriate risk perception may promote the improvement of patients' sense of disease benefit [20]. Zhang et al. found that accurate perception of recurrence risk in stroke patients is important for their further adoption of health behaviours, emotional regulation, and improved prognosis [21]. The above series of findings showed that risk perception has a direct impact on individual behaviour. The theory of risk perception and related interventions can be used to achieve individual behavior change.

From the perspective of risk perception, domestic and international scholars have conducted qualitative or semiquantitative research on risk in different fields. Quantitative research is relatively scarce, and there is no mention of physical subhealth risk perception measurement research. Combined with the above research results, we found that risk perception theory can be used for quantitative risk assessment in many fields and made a theoretical contribution to reduce the risk incidence. Graw et al. conducted a survey on the blood transfusion risk perception of 506 patients and 185 relevant medical staff in a German hospital [22]. The results showed that only 10.9% of patients and 14.6% of medical staff perceived the existence of blood transfusion risk. Based on this, we can believe that risk perception theory can be equally valuable in quantitative studies of physical subhealth and lay a foundation for studying the role of risk perception in subhealth early warning.

### 2.3. The Theory of Protective Action Decision Model (PADM)

The protective action decision model (PADM) is a multistage model that is based on findings from research on the responses of people to environmental hazards and disasters. The model identifies three core perceptions—threat perception, protective action perception, and stakeholder perception, which form the basis for decision-making on how to deal with threats [23]. PADM has been used to explain the process of people's perception of risk and explore how the public can take preventive measures to deal with risk events such as hurricanes, earthquakes, and floods [24–27], which can play a role in the construction of public subhealth risk perception. The PADM includes factors that induce individuals to take protective measures [23], so Cheng took Hefei as an example and used PADM to study the public's risk perception of increasingly serious city smog and the impact of city smog on citizens' protection behaviour [28]. We have reason to believe that PADM can also provide a theoretical framework for the construction of individual subhealth risk perception, which is helpful in the study of physical subhealth risk perception. Therefore, based on the theory of PADM, this study proposed a physical subhealth risk perception questionnaire and then developed and validated physical subhealth risk perception scale.

The public physical subhealth risk perception scale is a self-measurement tool for the public to prevent physical subhealth and provide a basis for future subhealth research. This study can improve the public's awareness of subhealth, increase the public's enthusiasm of subhealth screening, contribute to the prevention and timely intervention of public physical subhealth, and fill the gap in this research field in China.

## 3. Method

### 3.1. Initial Scale Development

As for the definition of physical subhealth risk perception, it can be considered as an individual's perception of the possibility of physical subhealth, that is, people's reaction to their own subhealth risk, which is an intuitive judgment. The risk perception model can be constructed with reference to PADM, covering three parts: threat perception, protection action perception, and physical health-related perception.

**Figure 1:**
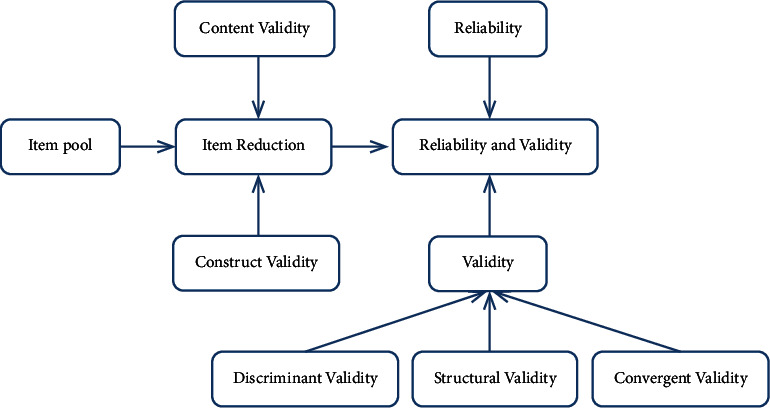
Research roadmap.

Learning from domestic and international scholars who have developed scales, we determined the purpose of the physical subhealth risk perception scale is to identify the level of public self-cognition of physical subhealth. In terms of threat perception, it is based on the understanding of subhealth, the level of awareness of subhealth, and the possible harm that subhealth can bring to an individual. In terms of protection action perception, it is based on the willingness to search for subhealth information, the channels to obtain subhealth information, and the processing methods of subhealth information. In terms of physical health-related perception, it is based on the benefits of paying attention to subhealth, the targets of subhealth information consultations, and the clinical symptoms of physical subhealth. Through literature research, we selected some items from the existing relevant questionnaires, combined with the experience of relevant scholars in constructing scales and the results of expert interviews to construct questionnaire items and finally form 90 initial measurement items. Through mechanism analysis, the above items were modified appropriately, and the overlapping information was separated to get the scale item pool, covering 56 items. In order to improve the content validity and construct validity of the items, we invited risk management experts, psychology expert, nursing expert, physician, and health management scholar to brainstorm the items of the item pool one by one, further clarified the structural relationship of measurement indicators, and formed the basic version of the scale, which mainly included 7 topics and 41 candidate items: (1) your understanding of subhealth knowledge (five items); (2) the benefits of physical examination (five items); (3) your understanding of subhealth (nine items); (4) the willingness to search for the information related to subhealth (five items); (5) the provider of accurate information about subhealth: doctors of local community hospitals, relatives, colleagues, etc. (six items); (6) the channels used to obtain the information related to subhealth: Internet search (e.g., Baidu and SOSO) and Social Network Service (e.g., QQ and WeChat) (six items); and (7) your handling of subhealth information (five items). On this basis, we carried out presurvey in 50 physical examinees from different regions. The participants ranged in age from 21 to 52, including 21 males and 29 females. Then, based on the results of the presurvey, we deleted and integrated some of the items and developed an initial physical subhealth risk perception scale, which mainly includes 7 dimensions, with a total of 35 items. The general scale is mostly scored in “Likert Scales.” Zhang suggested that the 3-grade scoring standard is somewhat restrictive in terms of the degree of expression of the participant's opinion, and that the scoring beyond 5-grade may affect the participant's ability in discrimination; thus, the 5-grade scoring standard is the most reliable method for expressing opinions and differentiating among items in the questionnaire [29]. Therefore, Likert's 5-grade scoring standard for each item was used in our study, and each item has five options, namely, “totally disagree, basically disagree, neither agree nor disagree, basically agree, and fully agree,” which scored 1-5 points, respectively. The higher the score he gets, the better the understanding of physical subhealth information he has, and also the higher the perception level of physical subhealth risk he has. The specific research roadmap is shown in Figure 1.

### 3.2. Participants

A random sampling method was used to develop the survey. A total of 35 items were included in the initial scale. A suitable sample size is considered to be 10 to 20 times the number of items [30]. The participants of the study were selected from the physical examination centres in Anhui, China. The survey time was from June to September 2019. 785 questionnaires were collected in this study. After eliminating the invalid questionnaires, 770 complete and valid questionnaires were obtained, with a valid response rate of 98%, meeting the sample requirements.

### 3.3. Available Criteria Tool

In order to explore the convergent validity of the target scale and carry out reliable empirical studies, based on the description of the clinical manifestations in terms of physiological functions in the Clinical Guidelines of Chinese Medicine on Subhealth issued by China Association of Chinese Medicine in 2006 [31], we collected 12 clinical manifestations of physical subhealth including the following symptoms: symptoms of short-term knee pain, progressive menopause, anxiety and depression, abnormal sleep, decreased flexibility, and susceptible to sickness and poor digestion; problems with skin, urine, pulse, and hair loss; body imbalance (e.g., pain, swelling, itching, cold, and similar symptoms); gastrointestinal and liver abnormalities (e.g., nausea in the morning, palpitations, and similar symptoms); abnormalities of the heart (e.g., shortness of breath, arrhythmia, snoring, sexual dysfunction, and similar symptoms); abnormal stool (e.g., alternating diarrhea and constipation and long-term chronic diarrhea); abnormal lack of pain (e.g. painless neck mass, painless hematuria, and similar symptoms); and other abnormalities (e.g., dizziness, dry throat, itching, pain, and edema of both eyes). Clinical manifestation options are divided into three categories: no, yes, and no idea.

### 3.4. Data Collection and Quality Control

The method of questionnaire survey was used to collect data. The questionnaire consisted of three sections: the first section on demographic characteristics, the second one on the available criteria tool (clinical manifestations of physical subhealth), and the third one on the physical subhealth risk perception scale. Before the survey, the investigators were trained to understand and be familiar with the standard of filling out the questionnaire. First, we obtained the informed consent of the participants; in order to save time for research participants, the verbal informed consent was obtained, and the questionnaire was distributed by the researcher on the spot. In case of doubt, the research participants can ask investigators face-to-face. If participants' response to each performance is unclear even after the researcher's explanations, then the participant will be removed to ensure the authenticity and accuracy of the results. It took about 10 minutes to complete the questionnaire. The participants volunteered to fill in the questionnaire. After completing the questionnaire, the researchers carefully checked whether the information on the questionnaire was complete. If there was any omission, the research participants were immediately asked to fill in the questionnaire completely. After verification, the questionnaires with obvious logic errors were excluded from the received questionnaires. This approval procedure was approved by the Ethics Committee of Anhui Medical University.

### 3.5. Statistical Analysis Method

The EpiData 3.1 software was used to establish the database, input data, and data cleaning. Firstly, the 35 items of the initial scale of physical subhealth risk perception were screened. The SPSS17.0 software was used to analyse the Pearson correlation of 35 items in the initial scale. Because items had an ordinal response scale, we conducted an exploratory factor analysis using a polychoric correlation matrix [32]. Common factors were extracted using the principal axis factoring method and the promax rotation method. Without imposing a fixed number of factors to extract, factors were extracted using the rule of eigenvalue ≥ 1. We used the resulting pattern coefficients, structure coefficients, and communality (shared variance) coefficients to create a Pratt matrix in which the *D* column (Pratt *D*) values are calculated by combining information from the pattern and structural factors [33], to partition the communality of each item (the proportion of variance that the item shares with other items) into nonoverlapping parts attributable to each factor. Pratt *D* measures the proportion of an item's communality explained by each factor. The higher the *D* value, the greater the proportion of the item's communality in each factor, and that means the contribution of the item is higher in the factor [34]. In theory, it should range from 0 (if none of the variance is explained by the factor) to 100 (if all of the variance is explained). In practice, values can sometimes be slightly negative or slightly greater than 1. We evaluated reducing the number of items by examining item-item correlations. If the correlation coefficient *r* > 0.6, the Pratt *D* values of the two items were calculated, respectively, and the Pratt *D* matrix was constructed by deleting the items with low Pratt *D* value.

Finally, reliability analysis and validity analysis were conducted on the screened items. We used the Cronbach'*α* coefficient method to test the internal consistency reliability of the scale [35]. It is generally believed that the closer the coefficient is to 1, the better the internal consistency and the higher the homogeneity reliability it has. The testing of validity included construct validity, discriminant validity, and convergent validity. Construct validity was examined by exploratory factor analysis and confirmatory factor analysis. Discriminant validity was tested by exploratory factor analysis, including principal component analysis and maximum variance rotation method [36–38]. The Amos 23.0 software was used for confirmatory factor analysis. Convergent validity was tested by using our general linear model.

## 4. Results

### 4.1. Descriptive Analysis

Among the 770 participants, there were 312 males and 458 females, mainly from second-tier cities (411) and towns (132), but also some from rural areas (117) and third-tier cities (110). The average age of the participants was 34 years old, with ages ranging from 18 to 79. The participants were generally highly educated, with 366 (47.5%) having a bachelor's degree or above. 499 (64.8%) perceived their health status as subhealth, 67 (8.7%) thought their health status was worse than subhealth, 61 (7.9%) did not know their health status, and only 143 (18.6%) thought they were healthy. There were significant differences among participants in terms of age, education level, living place, and self-assessment of health (*p* < 0.05), and there were no statistical differences in terms of gender (Table 1 for details).

### 4.2. Item Screening and Factor Analysis

**Figure 2:**
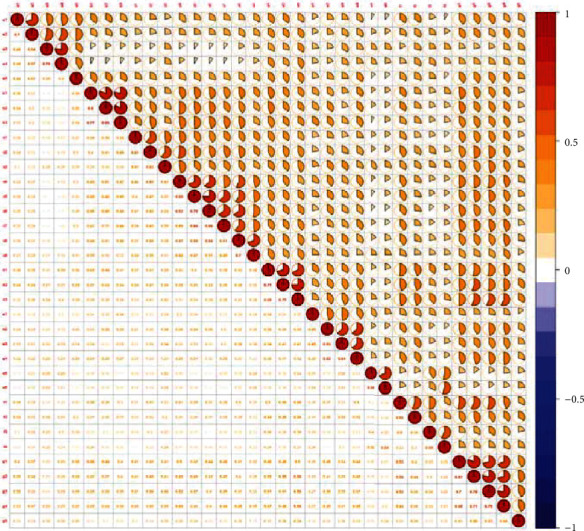
Project correlation matrix. Note: all data are significantly correlated at 0.01 level.

The correlation study of 35 items in the initial scale was conducted, and the results are shown in Figure 2 below. We can see from Figure 2 that the Pearson correlation coefficient ranges from 0.073 to 0.832 in all 35 items. The correlation coefficients between a1 and a2, a3 and a4, b1 and b2, b2 and b3, c4 and c5, c5 and c6, c6 and c7, c8 and c9, d1 and d2, d2 and d3, e5 and e6, g1 and g2, g2 and g3, and g2 and g4 were greater than 0.6.

We reduced the number of items by examining item-item correlations [32]. If the correlation coefficient between two items was greater than 0.6, the Pratt *D* values were calculated, respectively, and the items with low *D* values were deleted. Exploratory factor analysis was conducted on the 35 items, and Pratt *D* matrix was constructed by using the results of pattern coefficients, structure coefficients, and communality coefficients obtained from exploratory factor analysis (see Table 2 for the results).  Table 3 contains the calculation method for *D* values.

Based on the above research results, item a1 was highly correlated with item a2 (*r* = 0.70); the Pratt matrix *D* value was 0.83 for item a1 and 0.92 for item a2, so we dropped item a1. Item a3 was highly correlated with item a4 (*r* = 0.76); the Pratt matrix *D* value was 0.93 for item a3 and 0.90 for item a4, so we dropped item a4. For similar reasons, we deleted items b1, b3, c4, c5, c7, c8, d1, d3, e5, g1, g3, and g4 (see Table 2 for details). Because items b2, a5, and g5 had particularly low communality estimates of 0.41, 0.47, and 0.49, they were also dropped [32]. In total, we deleted 17 items.

Table 3 shows the Pratt *D* matrix of the remaining 18 items. The contribution of items was determined by *D* value in the Pratt *D* matrix [32]. Focusing on the rows of the Pratt *D* matrix allowed to compare the importance of the factors to the communality of each observed indicator (horizontal interpretation), whereas focusing on the columns of the Pratt *D* matrix allowed to compare the contribution of the indicators to the common variance extracted by each factor (vertical interpretation). Factor 1 included items c1, c2, c3, c6, and c9 (recorded as risk perception), and item c1 made the largest contribution; factor 2 included items e1, e2, e3, and e4 (recorded as trust selection), and items e2 and e3 made the largest contribution; factor 3 included items d2, f1, f2, and g2 (recorded as information channel), and item f1 made the largest contribution; factor 4 included items e6, f3, and f4 (recorded as social groups), and item f4 made the largest contribution; factor 5 included items a2 and a3 (recorded as health knowledge), and item a3 made the largest contribution. These factors were clearly distinct, having very little overlap. The correlation coefficients ranged from 0.19 to 0.51. The average score of the final scale was 66.9 ± 13.0, and the observation range was 18-90. Finally, the 18-item 5-factor scale shown in Table 3 was obtained (see Table 3).

### 4.3. Reliability Analysis and Validity Analysis

#### 4.3.1. Reliability Analysis

We further explored the Cronbach'*α* coefficient of the physical subhealth risk perception scale. The Cronbach'*α* coefficient of the scale was determined to be 0.889. The Cronbach'*α* coefficients of the five factors were 0.780, 0.825, 0.801, 0.736, and 0.704, respectively.

#### 4.3.2. Validity Analysis

We evaluated the validity of the physical subhealth risk perception scale from construct validity, discriminant validity, and convergent validity.

*(1) Construct Validity*. The total samples were randomly divided into sample 1 (*n* = 385) and sample 2 (*n* = 385) for exploratory factor analysis and confirmatory factor analysis, respectively.

The results of exploratory factor analysis showed that the Bartlett Sphericity test of the scale reached a significant level (*χ*^2^ = 2857.678, df = 153, and *p* < 0.001), indicating that it was suitable for factor analysis. The statistical value of KMO (Kaiser Meyer Olkin) was 0.863, which was greater than 0.5, indicating that the partial correlation between variables was very weak, and the effect of factor analysis was good. Five common factors with eigenvalues greater than 1 were retained, which were consistent with the factors of the scale obtained by the Pratt *D* matrix. The cumulative contribution rate of factors was 65.97%, and the factor load of each item was 0.503 to 0.826. In the rotated component matrix, it was found that the load values of item f3 on factor 3 and factor 4 are greater than 0.5, which should be deleted from the perspective of statistics. However, we decided to retain the item due to the perspective of professional and conceptual connotation.

In confirmatory factor analysis, the evaluation indicators included *χ*^2^/df, Root Mean Square Error of Approximation (RMSEA), Goodness-of-Fit Index (GFI), Normal-of-Fit Index (NFI), Relative Fit Index (RFI), and Comparative Fit Index (CFI). The results of the above evaluation indicators of the physical subhealth risk perception scale were *χ*^2^/df = 3.43, RMSEA = 0.08, GFI = 0.88, NFI = 0.84, RFI = 0.81, and CFI =0.88 (see Table 4).

*(2) Discriminant Validity*.  Table 5 was obtained by confirmatory factor analysis. It can be seen that the factors F1–F5 have significant correlation (*p* < 0.01), and the correlation coefficients are all less than the square root value of AVE. These results indicated that there is a certain correlation between the variables, and there is also a certain degree of discrimination between them [39, 40].

*(3) Convergent Validity*. To further verify the validity of the physical subhealth risk perception scale, we conducted an empirical study on the public perception of subhealth symptoms. We discussed the relationship between the clinical manifestations of physical subhealth and the level of risk perception, so as to evaluate the convergent validity of the physical subhealth risk perception scale. The clinical manifestations of physical subhealth include the following symptoms: symptoms of short-term knee pain (recorded as variable *a*); progressive menopause, anxiety, and depression (recorded as *b*); abnormal sleep (recorded as *c*); decreased flexibility (recorded as *d*); very susceptible to sickness and poor digestion (recorded as *e*); problems with skin, urine, pulse, and hair loss (recorded as *f*); body imbalance (e.g., pain, swelling, itching, cold, and similar symptoms) (recorded as *g*); gastrointestinal and liver abnormalities (e.g., nausea in the morning, palpitations, hunger, and similar symptoms) (recorded as *h*); abnormalities of the heart (e.g., shortness of breath, arrhythmia, snoring, sexual dysfunction, and similar symptoms) (recorded as *i*); abnormal stool (e.g., alternating diarrhea and constipation and long-term chronic diarrhea) (recorded as *j*); abnormal lack of pain (e.g., painless neck mass, painless hematuria, and similar symptoms) (recorded as *k*); and other abnormalities (e.g., dizziness, dry throat, itching, pain, and edema of both eyes) (recorded as *l*). The results of the general linear model are shown in Table 6.

We evaluated the convergent validity of the total scale and the five factors on the 12 subhealth clinical manifestations of the participants. From the above research results, we can see that the *p* values of all variables in the full scale are less than 0.05; only the *p* value of variable f is greater than 0.05, being 0.091. The *p* values indicated that the overall convergent validity of the scale is good. The convergent validity of variables *b*, *d*, *g*, and *l* on factor 1 was good (*p* < 0.05); variables *b*, *d*, *f*, *g*, *h*, *i*, *k*, and *l* had good convergent validity on factor 2 (*p* < 0.05); variables *a*, *d*, *e*, *h*, *j*, *k*, and *l* had good convergent validity on factor 3 (*p* < 0.05); variables *d*, *f*, *h*, *j*, and *k* had good convergent validity on factor 4 (*p* < 0.05), while variables *a*, *c*, *d*, *e*, *h*, *i*, *j*, and *k* had good convergent validity on factor 5 (*p* < 0.05).

## 5. Discussion

Our Pearson correlation study showed that the correlations among the 35 items were statistically significant, and the correlation coefficients between a1 and a2, a3 and a4, b1 and b2, b2 and b3, c4 and c5, c5 and c6, c6 and c7, c8 and c9, d1 and d2, d2 and d3, e5 and e6, g1 and g2, g2 and g3, and g2 and g4 were all greater than 0.6. The possible reason is the similarity between items. Furthermore, Pratt *D* matrix was used to analyse the multifactor structure to reduce the number of items and describe the factor structure. Finally, the physical subhealth risk perception scale with 18-item and 5-factor was obtained.

Reliability is mainly used to evaluate the accuracy, consistency, and stability of the scale and the variation degree of the measured value caused by random error in the measurement process. The Cronbach'*α* coefficient is usually used to measure the homogeneity or intrinsic correlation between items. When the research tool contains multiple items, the relationship between the items should be evaluated. It is considered that the Cronbach'*α* coefficient of the total scale should be greater than 0.8, and the Cronbach'*α* coefficient of each dimension should be greater than 0.6, which indicates high reliability [41, 42]. The Cronbach'*α* coefficient of the scale was 0.889, greater than 0.8, and the Cronbach'*α* coefficients of the five factors were 0.780, 0.825, 0.801, 0.736, and 0.704, respectively, all of which are greater than 0.6, indicating that the reliability of the scale was good. The internal consistency of each dimension was acceptable, suggesting that the scale had good internal consistency.

Factor analysis is an accepted method to evaluate the construct validity of the scale. In exploratory factor analysis, *χ*^2^/df is an indicator to evaluate the overall fitting degree of the model. *χ*^2^/df > 5 indicates that the overall fitting degree is poor; 3 < *χ*^2^/df < 5 indicates that the structural model is basically acceptable; *χ*^2^/df < 3 indicates that the overall structure fits well. The closer the *χ*^2^/df is to 0, the better the overall fit of the model is [43]. RMSEA ranges from 0 to 1. RMSEA < 0.10 indicates a good fit of the structural model [44]. The GFI, NFI, RFI, and CFI all range from 0 to 1, and the critical standard is 0.9, and the closer the fitting indicator is to 1, the better the model fit is [45]. The results of the fitting indicators in this study were as follows: the value of *χ*^2^/df was 3.43, indicating that the model fit was basically acceptable; the value of RMSEA was 0.08, which was less than 0.1, indicating that the fit of the structural model was ideal; the value of GFI was 0.88, below 0.9, but the value of GFI depends on the sample size [40]; the values of NFI and RFI were 0.84 and 0.81, respectively, which were less than 0.9, showing a relatively ordinary fit [40]. Based on the above indicators, the physical subhealth risk perception scale has an acceptable fit.

We compared the square root value of AVE with the correlation coefficient between factors to explore the discriminant validity of the scale. The square root value of AVE can represent the “convergence” of the factor, while the correlation coefficient represents the correlation relationship. If the factor itself has a strong “convergent,” that is, the square root value of AVE is larger than the correlation coefficient between this factor and other factors, it can show that the scale has discriminant validity. The results of confirmatory factor analysis showed that there were significant correlations among factors F1–F5 (*p* < 0.01), and the correlation coefficients were less than the corresponding square root value of AVE. These indicated that there was a certain correlation between the variables and a certain degree of discrimination between them, illustrating that the discriminant validity of the scale was ideal.

The results of our general linear model test showed that the *p* values of 11 of the 12 variables were less than 0.05, with the exception being variable *f* whose *p* value was 0.091. These *p* values indicated that the overall convergent validity of the scale was good. We also found that different variables have different convergent effectiveness at different factor levels. Based on the subhealth clinical manifestations of the participants, the general linear model was used to explore the convergent validity of the scale. The results showed significant differences in subhealth perception risk scores among participants with different physical subhealth clinical manifestations, which indicated that the scale passed the test of convergent validity.

The physical subhealth risk perception scale can be used in public physical subhealth risk measurement. The application of this new scale can enhance the public perception of subhealth. Positive risk perception attitudes of the public play a key role in driving individual adaptive behaviours and are a precondition for the public to make healthier lifestyle choices and participate into health screening. Populations with higher risk perception levels are more likely to resist unhealthy behaviours and adopt healthy lifestyle habits, which helps prevent the occurrence of physical subhealth. The construction of the scale is in line with the China's latest health policy of “Prevention First.” Early prevention can reduce the incidence rate of many diseases. The framed scale can be applied to the practice of early prevention. Its application in this area contributes considerably to the improvement of the quality of life for the public, which can save the national medical resources and provide insights for the formulation and implementation of the national health care policies.

## 6. Conclusion

Using the model on a real-world dataset, we developed and validated a reliable and valid 18 -item 5-factor physical subhealth risk perception scale; the Cronbach *α* coefficient of the scale was 0.889 and passed the validity test.

This new measure of physical subhealth risk perception is applicable to study the relationship between the level of physical subhealth risk perception and the clinical manifestations of physical subhealth. Previous studies have found that the higher the public's perception of risk, the more beneficial it is for the public to take measures to protect their own safety [46–48]. Similarly, improving the public's perception level of physical subhealth risk may also prompt the public to take measures to protect their own health, thus avoiding or delaying the occurrence of subhealth symptoms. Such changes are even conducive to the transformation of existing subhealth symptoms into healthy directions.

How to improve the public perception level of physical subhealth risk is an issue that needs attention in future research. The novel physical subhealth risk perception scale we present here had good reliability and validity on the dataset we used. We suggest an extended study, increasing the number of respondents and covering other parts of the public, to verify the appropriateness of the new scale. This confirmation of effectiveness should lead to using this scale to improve the overall health level of the public.

## Figures and Tables

**Table 1:** Characteristics of demographic factors of participants.

Variable	Number	Percentage (%)	Mean ± SD	*T*/*F*	*p*
Gender					
Male	312	40.5	1.59 ± 0.5	-0.99	0.323
Female	458	59.5			
Age/years					
<30	384	49.9	34.1 ± 11.6	4.07	0.007
30-45	256	33.2			
46-60	96	12.5			
>60	34	4.4			
Education					
Primary or below	44	5.7	3.97 ± 1.4	5.66	<0.001
Junior high school	103	13.4			
Senior high school	111	14.4			
Junior college	146	19.0			
Undergraduate	304	39.5			
Master's degree and above	62	8.1			
Living place					
Rural	117	15.2	3.06 ± 1.2	21.38	<0.001
Cities and towns	132	17.1			
Third-tier city	110	14.3			
Second-tier city	411	53.4			
Self-assessment of health					
Serious than subhealth	67	8.7	−0.64 ± 0.89	3.30	0.02
Subhealth	499	64.8			
Unclear	61	7.9			
Health	143	18.6			

**Table 2:** Items to be deleted.

Items	Correlation coefficient (*r*)	Pratt *D* value		Deleted item
a1 & a2	0.70	a1: 0.83	a2: 0.92	a1
a3 & a4	0.76	a3: 0.93	a4: 0.90	a4
b1 & b2	0.80	b1: 0.98	b2: 1.01	b1
b2 & b3	0.83	b2: 1.01	b3: 0.98	b3
c4 & c5	0.68	c4: 0.52	c5: 0.87	c4
c5 & c6	0.75	c5: 0.87	c6: 0.98	c5
c6 & c7	0.66	c6: 0.98	c7: 0.95	c7
c8 & c9	0.70	c8: 1.01	c9: 1.02	c8
d1 & d2	0.72	d1: 0.88	d2: 0.89	d1
d2 & d3	0.75	d2: 0.89	d3: 0.86	d3
e5 & e6	0.68	e5: 0.93	e6: 0.95	e5
g1 & g2	0.81	g1: 0.91	g2: 0.90	g1
g2 & g3	0.78	g2: 0.91	g3: 0.85	g3
g2 & g4	0.71	g2: 0.91	g4: 0.89	g4

**Table 3:** Pratt *D* matrix factor structure.

Item	*P*					*S*					*C*	*D*				
	F1	F2	F3	F4	F5	F1	F2	F3	F4	F5	_	F1	F2	F3	F4	F5
a2	0.04	-0.09	0.17	-0.10	0.85	0.33	0.31	0.40	0.14	0.86	0.77	0.02	-0.04	0.09	-0.02	0.95
a3	-0.07	0.03	-0.05	0.13	0.86	0.21	0.33	0.24	0.31	0.86	0.77	-0.02	0.01	-0.02	0.05	0.97
c1	0.84	-0.11	0.02	-0.03	-0.03	0.78	0.27	0.37	0.12	0.19	0.63	1.05	-0.05	0.01	-0.01	-0.01
c2	0.79	-0.08	0.20	0.02	-0.14	0.81	0.32	0.52	0.19	0.15	0.71	0.91	-0.04	0.15	0.01	-0.03
c3	0.66	0.04	0.02	0.19	-0.03	0.72	0.37	0.40	0.33	0.24	0.55	0.87	0.02	0.01	0.11	-0.01
c6	0.69	0.11	<0.01	-0.10	<0.01	0.72	0.41	0.36	0.06	0.23	0.53	0.93	0.09	<0.01	-0.01	<0.01
c9	0.64	0.03	-0.21	0.13	0.22	0.64	0.33	0.22	0.26	0.39	0.50	0.83	0.02	-0.09	0.07	0.17
d2	0.19	0.13	0.49	-0.11	0.16	0.52	0.47	0.66	0.12	0.40	0.53	0.18	0.11	0.61	-0.03	0.12
e1	-0.06	0.71	-0.02	0.26	-0.10	0.28	0.69	0.31	0.35	0.21	0.54	-0.03	0.91	-0.01	0.17	-0.04
e2	0.05	0.84	0.07	-0.09	-0.09	0.43	0.85	0.42	0.08	0.25	0.74	0.03	0.97	0.04	-0.01	-0.03
e3	-0.02	0.85	-0.03	0.03	0.08	0.38	0.87	0.37	0.20	0.40	0.76	-0.01	0.97	-0.01	0.01	0.04
e4	-0.03	0.74	0.20	-0.10	0.03	0.39	0.81	0.49	0.09	0.34	0.68	-0.02	0.87	0.14	-0.01	0.02
e6	0.15	0.15	-0.26	0.79	0.06	0.27	0.27	0.11	0.79	0.27	0.69	0.06	0.06	-0.04	0.90	0.02
f1	0.03	0.03	0.83	0.07	-0.09	0.44	0.39	0.85	0.29	0.22	0.73	0.02	0.01	0.97	0.03	-0.03
f2	-0.14	0.10	0.74	0.03	0.12	0.32	0.42	0.76	0.25	0.36	0.61	-0.07	0.07	0.92	0.01	0.07
f3	-0.11	-0.14	0.57	0.58	0.01	0.23	0.18	0.61	0.69	0.24	0.70	-0.03	-0.03	0.50	0.57	<0.01
f4	-0.01	-0.04	0.20	0.82	-0.03	0.23	0.19	0.39	0.86	0.22	0.77	<0.01	-0.01	0.10	0.92	-0.01
g2	0.29	0.05	0.60	-0.09	0.02	0.60	0.44	0.75	0.14	0.30	0.63	0.27	0.04	0.70	-0.02	0.01

Note: *C*: communality (the percentage of variance in each item explained by the 5 factors); *P*: pattern coefficient (the equivalent of the standardized partial regression coefficient, that is, the slope of a factor on the item); *S*: structure coefficient (the equivalent of the simple Pearson correlation between an item and each factor); *D* : *P*∗*S*/*C* (the Pratt *D* value, the proportion of the explained variance attributable to each factor; a measure of the relative importance of each factor to a given item); F1: factor 1; F2: factor 2; F3: factor 3; F4: factor 4; F5: factor 5. Factor 1 includes items c1, c2, c3, c6, and c9; factor 2 includes items e1, e2, e3, and e4; factor 3 includes items d2, f1, f2, and g2; factor 4 includes items e6, f3, and f4; factor 5 includes items a2 and a3.

**Table 4:** Scale fitting coefficient table.

*χ* ^2^/df	*p*	RMSEA	GFI	NFI	RFI	CFI
3.43	<0.001	0.08	0.88	0.84	0.81	0.88

**Table 5:** Discriminant validity: Pearson correlation and AVE square root value.

Factor	F1	F2	F3	F4	F5
F1	0.648				
F2	0.321^∗∗^	0.742			
F3	0.382^∗∗^	0.618^∗∗^	0.735		
F4	0.25^∗∗^	0.337^∗∗^	0.484^∗∗^	0.686	
F5	0.196^∗∗^	0.45^∗∗^	0.418^∗∗^	0.267^∗∗^	0.781

Note: the shaded part is the square root value of AVE extracted from the average variance; ^∗∗^ represents that the *p* < 0.01.

**Table 6:** Convergent validity.

Variable	No.	Full scale		Factor 1		Factor 2		Factor 3		Factor 4		Factor 5	
		Mean ± SD	*p*	Mean ± SD	*p*	Mean ± SD	*p*	Mean ± SD	*p*	Mean ± SD	*p*	Mean ± SD	*p*
*a*			0.001		0.006		0.475		0.037		0.316		0.034
No	364	67.9 ± 12.6		23.2 ± 4.7		15.0 ± 3.8		14.7 ± 3.7		9.4 ± 3.0		5.6 ± 2.0	
Yes	370	66.2 ± 12.9		22.7 ± 4.7		14.8 ± 4.0		13.9 ± 3.7		9.3 ± 3.0		5.5 ± 2.1	
No idea	36	63.5 ± 17.5		21.7 ± 6.1		13.2 ± 4.1		13.8 ± 4.3		9.7 ± 3.6		5.1 ± 2.3	
*b*			<0.001		0.004		0.019		0.460		0.396		0.228
No	258	65.8 ± 12.8		22.6 ± 4.8		14.7 ± 3.9		14.1 ± 3.8		8.9 ± 2.9		5.5 ± 2.0	
Yes	459	67.5 ± 12.8		23.1 ± 4.6		14.9 ± 3.9		14.4 ± 3.7		9.5 ± 3.0		5.6 ± 2.0	
No idea	53	67.4 ± 16.0		22.7 ± 5.8		14.7 ± 4.4		14.3 ± 3.9		10.3 ± 3.0		5.4 ± 2.3	
*c*			0.007		0.593		0.196		0.293		0.066		0.018
No	309	66.1 ± 12.6		22.6 ± 4.8		14.1 ± 3.7		14.1 ± 3.7		9.1 ± 3.0		5.3 ± 2.0	
Yes	405	67.5 ± 13.5		23.1 ± 4.7		14.7 ± 3.9		14.4 ± 3.8		9.6 ± 3.0		5.7 ± 2.0	
No idea	56	67.5 ± 12.3		14.7 ± 3.9		14.7 ± 3.6		14.3 ± 3.3		9.5 ± 2.9		5.9 ± 2.1	
*d*			<0.001		<0.001		0.005		0.002		0.045		0.006
No	347	67.0 ± 12.3		23.0 ± 4.8		14.7 ± 4.8		14.5 ± 3.5		9.4 ± 3.1		5.4 ± 1.9	
Yes	371	67.1 ± 13.7		23.0 ± 4.7		15.0 ± 4.1		14.2 ± 3.9		9.3 ± 3.0		5.6 ± 2.1	
No idea	52	65.4 ± 13.2		22.3 ± 4.7		14.2 ± 3.5		13.3 ± 3.8		9.6 ± 2.7		6.0 ± 2.1	
*e*			<0.001		<0.001		0.253		0.017		0.205		0.040
No	306	66.7 ± 12.5		23.0 ± 4.7		14.9 ± 3.9		14.1 ± 3.7		9.3 ± 3.0		5.5 ± 2.1	
Yes	417	67.6 ± 13.1		23.1 ± 4.6		14.9 ± 3.9		14.5 ± 3.7		9.5 ± 3.0		5.6 ± 2.0	
No idea	47	62.2 ± 15.0		21.0 ± 5.6		13.3 ± 3.7		13.0 ± 3.6		9.2 ± 3.0		5.7 ± 1.8	
*f*			0.091		0.244		0.001		0.312		0.043		0.413
No	264	66.6 ± 12.6		22.8 ± 4.8		14.7 ± 4.2		14.3 ± 3.8		9.2 ± 3.1		5.7 ± 2.0	
Yes	448	67.1 ± 13.0		23.0 ± 4.5		14.9 ± 3.8		14.2 ± 3.7		9.5 ± 2.9		5.5 ± 2.0	
No idea	58	66.8 ± 15.4		22.7 ± 6.0		14.8 ± 3.9		14.4 ± 4.1		9.5 ± 3.2		5.5 ± 2.4	
*g*			<0.001		0.408		0.001		0.667		0.410		0.074
No	302	66.8 ± 12.5		23.1 ± 4.6		14.7 ± 4.0		14.2 ± 3.8		9.2 ± 3.2		5.6 ± 2.1	
Yes	416	67.0 ± 13.0		22.9 ± 4.6		14.9 ± 3.9		14.3 ± 3.6		9.4 ± 2.9		5.6 ± 2.0	
No idea	52	67.4 ± 16.0		22.5 ± 6.1		15.1 ± 3.5		14.4 ± 4.1		10.±3.2		5.2 ± 2.2	
*h*			<0.001		0.472		0.005		0.002		0.041		0.001
No	287	65.3 ± 13.4		22.7 ± 5.0		14.5 ± 4.2		13.8 ± 4.0		9.0 ± 3.0		5.4 ± 2.1	
Yes	428	68.2 ± 12.1		23.2 ± 4.4		15.1 ± 3.7		14.7 ± 3.4		9.5 ± 3.0		5.7 ± 1.9	
No idea	55	65.2 ± 16.4		22.1 ± 6.0		14.3 ± 3.8		13.4 ± 4.1		9.7 ± 3.0		5.6 ± 2.4	
*i*			0.002		0.076		0.014		0.466		0.292		0.002
No	293	66.9 ± 13.1		23.0 ± 4.9		15.0 ± 4.3		14.2 ± 3.8		9.2 ± 3.3		5.6 ± 2.2	
Yes	426	66.9 ± 13.0		23.0 ± 4.6		14.7 ± 3.7		14.3 ± 3.7		9.4 ± 2.9		5.6 ± 1.9	
No idea	51	66.9 ± 13.6		22.5 ± 5.3		14.8 ± 4.3		14.3 ± 3.7		9.7 ± 2.7		5.6 ± 2.1	
*j*			<0.001		0.737		0.166		0.004		0.047		0.012
No	290	65.1 ± 12.4		22.6 ± 4.7		14.4 ± 4.0		13.7 ± 4.0		8.9 ± 2.9		5.5 ± 2.1	
Yes	429	68.5 ± 12.8		23.3 ± 4.5		15.2 ± 3.8		14.7 ± 3.5		9.7 ± 3.0		5.7 ± 1.9	
No idea	51	63.6 ± 16.8		21.7 ± 6.4		14.1 ± 4.3		13.6 ± 3.8		9.3 ± 3.5		4.8 ± 2.4	
*k*			0.008		0.231		0.001		0.018		0.002		0.002
No	237	65.6 ± 12.7		22.6 ± 4.5		14.6 ± 4.3		13.9 ± 4.0		9.0 ± 2.9		5.6 ± 2.2	
Yes	451	68.4 ± 12.4		23.3 ± 4.5		15.1 ± 3.7		14.7 ± 3.4		9.6 ± 3.0		5.7 ± 1.9	
No idea	82	62.4 ± 15.7		22.0 ± 6.1		13.8 ± 4.1		12.7 ± 4.3		9.1 ± 3.3		4.8 ± 2.0	
*l*			0.001		0.026		0.019		0.039		0.198		0.134
No	347	66.8 ± 12.4		23.1 ± 4.7		14.7 ± 3.8		14.2 ± 3.7		9.3 ± 2.9		5.4 ± 2.0	
Yes	382	67.1 ± 13.0		22.7 ± 4.5		14.9 ± 4.0		14.4 ± 3.7		9.4 ± 3.0		5.7 ± 2.0	
No idea	41	66.7 ± 18.4		23.0 ± 6.7		14.2 ± 4.4		13.7 ± 4.3		10.±3.7		5.7 ± 2.2	

Note: the *p* value was calculated by the general linear model to illustrate the clustering degree of the research participants.

## Data Availability

The data used to support the findings of this study are included within the article.

## Abbreviations

RMSEA: Root Mean Square Error of Approximation
GFI: Goodness-of-Fit Index
NFI: Normal-of-Fit Index
CFI: Comparative Fit Index
RFI: Relative-of-Fit Index
AVE: Average variance extraction
PADM: The theory of protective action decision model.

## Supplementary Materials

Supplementary 1 S1 File: subhealth risk perception scale (S-HRPS).

Supplementary 2 S2 File: subhealth risk perception measurement questionnaire (S-HRPMQ).
