# Supplementary material for: Subhealth Risk Perception Scale: Development and Validation of a New Measure
Source: Comput Math Methods Med. 2022 Jan 10;2022:9950890. doi: 10.1155/2022/9950890 (PMC8764275; doi:10.1155/2022/9950890)
Supplement: Supplementary 1 — S1 File: subhealth risk perception scale (S-HRPS). [file 9950890.f1.pdf]

Sub-health Risk Perception Measurement Questionnaire

1. Investigation questionnaire The following are single-choice questions, according to the above material, you agree with the options with a tick , to indicate the degree of your agreement or disagreement, of which completely disagree 1, basically disagree 2, neither agree nor disagree 3, basically agree 4, completely agree 5.

a	Your understanding of sub-health knowledge	Totally disagree ⟷ Totally agree				
a1	I know about sub-health/unhealthy	1	2	3	4	5
a2	I'm more knowledgeable about sub-health/unhealthy than the people around me	1	2	3	4	5
a3	I regularly browse and read health newsletters/exam related websites/sub-health related brochures	1	2	3	4	5
a4	I spend more time learning about sub-health/unhealthy than the people around me	1	2	3	4	5
a5	I am usually the primary decision maker when deciding whether my family will participate in health screenings	1	2	3	4	5
**b**	**The benefits of physical examination to physical health**	**Totally disagree ⟷ Totally agree**				
b1	It can detect abnormal health indicators in time for early intervention and reduce health risks.	1	2	3	4	5
b2	It can prevent major illnesses and reduce financial losses to families.	1	2	3	4	5
b3	Major diseases can be detected early, to prolong life	1	2	3	4	5
**c**	**Your understanding of sub-health**	**Totally disagree ⟷ Totally agree**				
c1	Total presence of sub-health/unhealthy indicators in an individual's body	1	2	3	4	5
c2	Sub-healthy/unhealthy physical symptoms that I fear are a threat to my quality of life	1	2	3	4	5
c3	Sub-health/unhealthy symptoms in my body and I feel anxious and scared	1	2	3	4	5
c4	Bad moods and emotions increase the probability of sub-optimal health/unhealthy findings	1	2	3	4	5
c5	Do you think the occurrence of sub-health/unhealthy is related to the individual's lifestyle?	1	2	3	4	5
c6	Do you think the occurrence of sub-health/unhealthy is related to the individual's behavioral habits?	1	2	3	4	5

**c7**	Do you think the occurrence of sub-health/unhealthy is related to the nature of the individual's work?	1	2	3	4	5
**c8**	Do you think the occurrence of sub-health/unhealthy is related to the degree of family harmony in an individual?	1	2	3	4	5
**c9**	Do you think the occurrence of sub-health/unhealthy is related to the degree of integrity of an individual's family structure?	1	2	3	4	5
**d**	**Whether you are willing to search for sub-health related information**	**Totally disagree ←→ Totally agree**				
**d1**	I'm willing to search for as much information as possible about sub-health/unhealthy often	1	2	3	4	5
**d2**	I need to search for more information about sub-health/unhealthy	1	2	3	4	5
**d3**	I would search for information about sub-health/unhealthy from more sources	1	2	3	4	5
**e**	**Who do you think can provide accurate information about sub-health: doctors of local community hospitals, relatives, colleagues, etc**	**Totally disagree ←→ Totally agree**				
**e1**	Doctors at local community hospitals	1	2	3	4	5
**e2**	Doctors in provincial and municipal hospitals	1	2	3	4	5
**e3**	Provincial or national public health administrators	1	2	3	4	5
**e4**	Experts/scholars at medical research institutions	1	2	3	4	5
**e5**	Friends, relatives, neighbors and colleagues	1	2	3	4	5
**e6**	Family members	1	2	3	4	5
**f**	**What channels do you use to obtain sub-health related information: internet search (e.g., Baidu, SOSO), Social Network Service(e.g., QQ, WeChat) (four items)**	**Totally disagree ←→ Totally agree**				
**f1**	Internet search (Baidu, Soso, etc.)	1	2	3	4	5
**f2**	Related Hospital Websites	1	2	3	4	5
**f3**	Social networks (QQ, WeChat, Weibo, etc.)	1	2	3	4	5
**f4**	Friends, relatives, neighbors and colleagues	1	2	3	4	5
**g**	**Your treatment of sub-health information**	**Totally disagree ←→ Totally agree**				
**g1**	I'll link it to information I've learned elsewhere	1	2	3	4	5
**g2**	I will compare this information with other relevant information	1	2	3	4	5
**g3**	I'll try to think about the importance of this information to me	1	2	3	4	5

**g4**	I would try to relate this information to quality of life	1	2	3	4	5
**g5**	I will do the presupposition scene with people living with a history of sub-health/unhealthy	1	2	3	4	5

2. Fill in the following items according to your personal situation: personal information (single choice or fill in the blank, anonymously, never disclose)

1 . Gender : ______________ A.Male B.Female

2 . Age : _____________

3 . Education level : ________

A.Primary school or below B.Junior high school

C.High school D.Post-secondary E.Undergraduate

F.Master's degree and above

4. Number of years you have worked :

A. < 5 B. 5~10 C. 11~20 D. 21~30 E. > 30

5. Job : A.Operator B. Managerial staff C.Technical staff D.Other

6. Where you live : A.Rural B.Cities and towns C.Third-tier city D.Second-tier city E.First-tier city

7. Your marital status : A. Unmarried B. Married C. Remarried D. Divorced E. Widower/Widow

8. Number of children : A. 0 B. 1 C.2 D. ≥3

9. Annual household income : _________________

A. Less than 30,000 yuan B. 30,000 to 60,000 yuan C. 60,000 to 100,000 yuan D. 100,000 to 200,000 yuan E. More than 200,000 yuan

10.Do you feel that your body is in a state of sub-health ?

A. Health B. Sub-health C. More serious than sub-health D. Unclear

11.If you were in the state of sub-health, how long did it last? ? ( )

A. Less than 3 months B. 3 to 6 months C. 6 months to 1 year

D. Less than 2 years E. More than 3 years

12.Number of people in your workplace :

A. Freelance B. Less than 50 C. 50 to 150 D.150 to 500 E. ≥500

13. You feel that the percentage of the colleagues in your organization who are in sub-health is ( )

A. 10% B. 30% C. 50% D. 70% E. ≥90%
