# Supplementary material for: Subhealth Risk Perception Scale: Development and Validation of a New Measure
Source: Comput Math Methods Med. 2022 Jan 10;2022:9950890. doi: 10.1155/2022/9950890 (PMC8764275; doi:10.1155/2022/9950890)
Supplement: Supplementary 2 — S2 File: subhealth risk perception measurement questionnaire (S-HRPMQ). [file 9950890.f2.pdf]

Sub-health Risk Perception Scale

The following are single-choice questions, according to the above material, you agree with the options on the tick, to indicate the degree of your agreement or disagreement, of which completely disagree 1, basic disagree 2, neither agree nor disagree 3, basic agree 4, completely agree 5.

	**Health knowledge**	**Totally disagree ←→ Totally agree**				
	I'm more knowledgeable about sub-health/unhealthy than the people around me	1	2	3	4	5
	I regularly browse and read health newsletters/exam related websites/sub-health related brochures	1	2	3	4	5
	**Trust selection**	**Totally disagree ←→ Totally agree**				
	Doctors at local community hospitals	1	2	3	4	5
	Doctors in provincial and municipal hospitals	1	2	3	4	5
	Provincial or national public health administrators	1	2	3	4	5
	Experts/scholars at medical research institutions	1	2	3	4	5
	**Information channel**	**Totally disagree ←→ Totally agree**				
	Internet search (Baidu, Soso, etc.)	1	2	3	4	5
	Related Hospital Websites	1	2	3	4	5
	I need to search for more information about sub-health/unhealthy	1	2	3	4	5
	I will compare this information with other relevant information	1	2	3	4	5
	**Risk perception**	**Totally disagree ←→ Totally agree**				
	Total presence of sub-health/unhealthy indicators in an individual's body	1	2	3	4	5
	Sub-healthy/unhealthy physical symptoms that I fear are a threat to my quality of life	1	2	3	4	5
	Sub-health/unhealthy symptoms in my body and I feel anxious and scared	1	2	3	4	5

	Do you think the occurrence of sub-health/unhealthy is related to the individual's behavioral habits?	1	2	3	4	5
	Do you think the occurrence of sub-health/unhealthy is related to the degree of integrity of an individual's family structure?	1	2	3	4	5
	**Social groups**	**Totally disagree ←→ Totally agree**				
	Family members	1	2	3	4	5
	Social networks (QQ, WeChat, Weibo, etc.)	1	2	3	4	5
	Friends, relatives, neighbors and colleagues	1	2	3	4	5
